# A novel predicted ADP-ribosyltransferase-like family conserved in eukaryotic evolution

**DOI:** 10.7717/peerj.11051

**Published:** 2021-03-10

**Authors:** Zbigniew Wyżewski, Marcin Gradowski, Marianna Krysińska, Małgorzata Dudkiewicz, Krzysztof Pawłowski

**Affiliations:** 1 Institute of Biological Sciences, Cardinal Stefan Wyszynski University in Warsaw, Warszawa, Poland; 2 Department of Biochemistry and Microbiology, Warsaw University of Life Sciences - SGGW, Warszawa, Poland; 3 Department of Molecular Biology, University of Texas Southwestern Medical Center, Dallas, TX, United States; 4 Department of Translational Medicine, Lund University, Lund, Sweden

**Keywords:** ADP-ribosyltransferases, Evolution, Protein domains, Pseudoenzymes, Protein structure and function prediction

## Abstract

The presence of many completely uncharacterized proteins, even in well-studied organisms such as humans, seriously hampers full understanding of the functioning of the living cells. ADP-ribosylation is a common post-translational modification of proteins; also nucleic acids and small molecules can be modified by the covalent attachment of ADP-ribose. This modification, important in cellular signalling and infection processes, is usually executed by enzymes from the large superfamily of ADP-ribosyltransferases (ARTs). Here, using bioinformatics approaches, we identify a novel putative ADP-ribosyltransferase family, conserved in eukaryotic evolution, with a divergent active site. The hallmark of these proteins is the ART domain nestled between flanking leucine-rich repeat (LRR) domains. LRRs are typically involved in innate immune surveillance. The novel family appears as putative novel ADP-ribosylation-related actors, most likely pseudoenzymes. Sequence divergence and lack of clearly detectable “classical” ART active site suggests the novel domains are pseudoARTs, yet atypical ART activity, or alternative enzymatic activity cannot be excluded. We propose that this family, including its human member LRRC9, may be involved in an ancient defense mechanism, with analogies to the innate immune system, and coupling pathogen detection to ADP-ribosyltransfer or other signalling mechanisms.

## Introduction

ADP-ribosyltransferases (ARTs) are enzymes catalyzing the transfer of ADP-ribose from oxidized nicotinamide adenine dinucleotide (NAD^+^) to different acceptor molecules. Thus, they are responsible for chemical modification of various targets such as proteins, nucleic acids and small molecules. These enzymes are highly conserved in evolution and widespread in nature. They are common to all three domains of life: the Archaea, the Bacteria and the Eukarya. Multiplicity of ADP-ribosylated substrates is reflected in a variety of functions performed by ARTs (Aravind et al., 2015; Cohen & Chang, 2018; Munnur & Ahel, 2017; Otto et al., 2005; Teloni & Altmeyer, 2016).

Prokaryotic ADP-ribosyltransferases often play a role of bacterial toxins or effectors. Several studies show that ADP-ribosylation of the host target molecules by bacterial ARTs is correlated with the development of infection (Aktories et al., 2011; Bhogaraju et al., 2016; Boyer et al., 2006; Kalayil et al., 2018; Simon, Aktories & Barbieri, 2014). At the intracellular scale, ADP-ribosylation may change apoptotic potential of infected cell (Klockgether & Tümmler, 2017) and/or disturb organization of cellular membranes and actin cytoskeleton (Kagan & Roy, 2002; Komander & Randow, 2017; Maresso et al., 2007) whereas at systemic level, it can disturb cell-mediated immune response (Bhogaraju et al., 2016; Klockgether & Tümmler, 2017), or increase the permeability of barriers limiting bacterial spread (epithelium and endothelium) (Boyer et al., 2006; Klockgether & Tümmler, 2017) and consequently contribute to serious structural and functional disorders of the host tissues and organs (Chiu et al., 2009; Munro et al., 2010). Some ARTs act on small molecules, e.g., the bacterial Arr enzymes that ADP-ribosylate the antibiotic rifamycin (Baysarowich et al., 2008).

Eukaryotic ADP-ribosyltransferases play important roles in both physiological and pathophysiological processes, contributing to changes in chemical properties of the ADP-ribose acceptors. Proteins, post-translationally modified by ARTs, can also lose the capacity to interact with their ligands or acquire the ability to bind new ones. ADP-ribosylation of enzymes may cause substantial changes in their catalytic activity (Jwa & Chang, 2012; Liu & Yu, 2015). ADP-ribose moieties may function as signals that direct modified acceptors to ubiquitin-dependent proteolysis (Aravind et al., 2015; Cohen & Chang, 2018). Influencing the half-life of the target molecules, ADP-ribosyltransferases determine their intracellular level and thus affect their activity (Cohen & Chang, 2018). Moreover, ADP-ribose moieties may form molecular scaffolds with a negative charge. Such structures play a role in recruitment of positively charged proteins, favoring specific intermolecular interactions (Leung et al., 2012). Poly-ADP-ribosylation of DNA-binding proteins (e.g., core histones (Javle & Curtin, 2011; Weaver & Yang, 2013), chromatin remodeling enzymes (e.g., histone demethylase KDM5B (Krishnakumar & Kraus, 2010), DEK protein (Gamble & Fisher, 2007) and DNA repair factors (Kim et al., 2015) influences genome organization (Weaver & Yang, 2013) and expression (Gamble & Fisher, 2007; Krishnakumar & Kraus, 2010) as well as effective DNA repair (Javle & Curtin, 2011; McCann, 2019). Recently, growing evidence shows that PARPs also directly ADP-ribosylate mRNA (Kim et al., 2020). Other examples of nucleic acid ADP-ribosylation are provided by the bacterial toxin DarT that acts on ssDNA (Lawaree et al., 2020) and the toxin scabin, acting on mononucleosides, nucleotides, and both single-stranded and dsDNA (Lyons et al., 2018).

The members of the poly-ADP-ribosyltransferase (PARP) family, PARP1 and PARP2, are examples of ARTs that modify proteins interacting with DNA. PARP1 and PARP2 are localized in the nucleus where they are involved in many cellular processes (Choi et al., 2016; Liang et al., 2013; Riccio, Cingolani & Pascal, 2016; Szántó et al., 2014). PARP1 and PARP2 are engaged in DNA repair, that is elimination of DNA damage such as deamination, hydroxylation and methylation as well as for repair of single strand breaks. PARP1 and other PARPs also play a role in maintaining telomere stability (Bai, 2015; De Vos, Schreiber & Dantzer, 2012). PARPs are known as EMA/FDA approved drug targets, PARP inhibitors are used in treatment of prostate, breast and ovarian cancers. (Curtin, 2005; Curtin & Szabo, 2013; Kamel et al., 2018; Kummar et al., 2012).

In general, ADP-ribosyltransferases are responsible for regulation of intracellular and extracellular signal transduction. Therefore, their activity determines viability and proliferation potential of cells, DNA stability, immune system reactivity and thus the proper functioning of eukaryotic organisms (Bai, 2015; Corda & Di Girolamo, 2002; De Vos, Schreiber & Dantzer, 2012; Yamamoto et al., 2017). On the other hand, ADP-ribosyltransferases may also be involved in development of pathological states such as neurodegenerative disorders, diabetes, atherosclerosis, cataract and cancer (Barreiro & Gea, 2018; Gunderson & Moore, 2015; Kamboj et al., 2013; Malyuchenko et al., 2015; Mangerich & Bürkle, 2012; Rossi, Ghosh & Bohr, 2010). Similarly to protein phosphorylation, ADP-ribosylation is a reversible post-translational modification, performed by a trio of “writers” (ARTs), “readers” (e.g., macro domain proteins) and “erasers” (e.g., some macro domains, ADP-ribose hydrolases, and NUDIX phosphodiesterases) (O’Sullivan et al., 2019).

**Table 1:** List of known human ART-like domains. Full list of ART-like domains from human, including the novel DUF3715 domains in TASOR, TASOR2 and TEX15.

**Name**	Gene names*	**family classification for ART Domain (Pfam)**		**Triad motif**	**R/H-G-T/S motif in β-strand 1**	**S-X-S/Y-X-X motif in β-strand 2**	**X-X-E motif at front edge of β-strand 5**	**ADP-ribosylationactivity: mono (M), oligo (O), poly (P)**	**Cellular localization**	**ADP-ribosylation target: protein (P), DNA (D), RNA (R), and auto-ADP- ribosylation (A)**	**Total length (ART-like domain length)**
TRPT1	TRPT1	PTS_2-RNA	PF01885	H-H-V	HGT	HLA	NGV	M	mitochondrion, nucleus, endoplasmic reticulum, cytosol	R	253 (181)
ART1	ART1	ART	PF01129	R-S-E	RGV	SAS	EEE	M	sarcoplasmic reticulum membrane, plasma membrane, ER membrane and lumen, glycosylphosphatidylinositol (GPI)-anchored ectoenzymes	P	327 (222)
ART3	ART3 TMART	ART	PF01129	K-L-V	RTS	SAK	ERI	M	cell membrane, extracellular region, extracellular exosome	P	389 (222)
ART4	ART4 DO DOK1	ART	PF01129	G-S-E	YRT	STS	KKE	M	cell membrane, extracellular region, extracellular exosome, glycosylphosphatidylinositol (GPI)-anchored proteins	P	314 (222)
ART5	ART5 UNQ575/PRO1137	ART	PF01129	R-S-E	RGV	SSS	ERE	M	secreted	P	291 (226)
PARP1	**PARP1 ADPRT PPOL**	PARP	PF00644	H-Y-E	HGS	YFA	YNE	P	nucleus	D, P	1014 (227)
PARP2	**PARP2 ADPRT2ADPRTL2**	PARP	PF00644	H-Y-E	HGS	YFA	YNE	P	nucleus	D, P, A	583 (228)
PARP3	**PARP3 ADPRT3ADPRTL3**	PARP	PF00644	H-Y-E	HGT	YFA	QSE	M	nucleus	D, P, A	533 (221)
PARP4	**PARP4 ADPRTL1KIAA0177 PARPL**	PARP	PF00644	H-Y-E	HGS	YFS	DDE	M	nucleus, exosomes, cell membrane, spindle	P	1724 (205)
PARP5A (tankyrase-1)	TNKS PARP5A PARPL TIN1 TINF1 TNKS1	PARP	PF00644	H-Y-E	HGS	YFA	YAE	O, P	nucleus, telomeres, Golgi apparatus, cytoplasm	P, A	1327 (206)
PARP5B (tankyrase-2)	TNKS2 PARP5B TANK2 TNKL	PARP	PF00644	H-Y-E	HGS	YFA	LAE	O, P	nucleus, telomeres, Golgi apparatus, cytoplasm	P, A	1166 (206)
PARP6	PARP6	PARP	PF00644	H-Y-I	HGS	YLS	GEI	M	cytoplasm	P, A	630 (227)
PARP8	PARP8	PARP	PF00644	H-Y-I	HGS	YLS	GNI	M	nucleus, endoplasmic reticulum	P, A	854 (228)
PARP16	PARP16 ARTD15 C15orf30	PARP	PF00644	H-Y-Y	HGS	YLT	PKY	M	cell membrane, endoplasmic reticulum	P, A	322 (186)
PARP15	* PARP15 BAL3*	PARP	PF00644	H-Y-L	HGT	YFA	PKL	M	cytoplasm	R, P, A	678 (197)
PARP14	* PARP14 BAL2 KIAA1268*	PARP	PF00644	H-Y-L	HGT	YFA	PSL	M	cytoplasm, nucleus	P, A	1801 (197)
PARP10	* PARP10*	PARP	PF00644	H-Y-I	HGT	YFA	PSI	M	nucleus, cytoplasm	R, P, A	1025 (220)
PARP13	*ZC3HAV1 ZC3HDC2 PRO1677*	PARP	PF00644	Y-Y-V	YAT	YFA	PSV	inactive	cytoplasm	–	902 (187)
PARP7	*TIPARP PARP7*	PARP	PF00644	H-Y-I	HGT	YFA	PQI	M	nucleus, cytoplasm	P, A	657 (209)
PARP12	*PARP12 ZC3HDC1*	PARP	PF00644	H-Y-I	HGT	YFA	PSI	M	cytoplasm	P, A	701 (215)
PARP11	*PARP11 C12orf6*	PARP	PF00644	H-Y-I	HGT	YFA	PKI	M	nucleus (in mice)	R, P, A	338 (216)
PARP9	* PARP9 BAL, BAL1*	Undetected **/insignificant		Q-Y-T	QQV	YFT	PET	M	nucleus, cell membrane, cytoplasm, mitochondrion	ubiquitin	854 (223)
**TASOR**	TASOR	DUF3715 (partial alignment)	PF12509	L-Y-Q	LMV	YLS	LTQ	inactive	nucleus, chromosome	–	1670 (221)
**TASOR2**	TASOR2	DUF3715 (partial alignment)	PF12509	*V-T-E*	*VVA*	*TLD*	*LLE*	not determined	cytoplasm, nucleoplasm	not determined	2430 (199)
**TEX15**	TEX15	DUF3715 (partial alignment)	PF12509	*L-Y-S*	*LAL*	*YMF*	*VLS*	not determined	nucleus, cytoplasm	not determined	2789 (197)
**NEURL4**	NEURL4	Undetected**		*H-L-E*	*HGS*	*LLS*	*ELE*	not determined	cytoplasm, cytoskeleton	not determined	1562 (121)
**LRRC9**	LRRC9	Undetected**		*Y-V-K*	*YVF*	*EFL (SIS)*	*QCK*	not determined	not determined	not determined	1453 (233)

**Notes.**

* PARP family members are sorted by subgroup (font format) on the basis of similarities in amino acid sequence, intron positions and associated protein domains (Otto et al., 2005).

** For these domains, Pfam family assignments cannot be made using standard Pfam HMM tool.

Italicized motifs were identified based on HHpred and FFAS03 alignments to canonical PARPs, in some cases the two methods were not in agreement.DUF3715 domains correspond to a part of TASOR-like ART domains.

According to the Pfam database, the ART clan (superfamily) comprises 23 families of domains, fourteen of which, ADPrib_exo_Tox, ART, DarT, DUF2441, DUF3990, DUF952, Enterotoxin_a, Exotox-A_cataly, PARP, Pertussis_S1, PTS_2-RNA, RES, RolB_RolC and TNT, include eukaryotic members (El-Gebali et al., 2019). However, some of these 14 families are predominantly bacterial with just a handful of eukaryotic members, e.g. ADPrib_exo_Tox and DarT. PARPs, the best studied family of ADP-ribosyltransferases, are responsible for modification of target structures by covalently adding polymeric chains of ADP-ribose moieties instead of transferring only one moiety. Despite low conservation of amino acid sequence, ART clan members are characterized by a common spatial structure comprising a split β-sheet and two helical regions surrounding it. The “split” separates the β-sheet into two halves, each composed of three β-strands (4-5-2 and 1-3-6, respectively) (Aravind et al., 2015; Cohen & Chang, 2018). Aravind and colleagues divide ARTs into three main clades: the H-H-h clade, the H-Y-[EDQ] clade and the R-S-E clade (Aravind et al., 2015; Cohen & Chang, 2018) that are characterized by the different configurations of active site amino acid residues. In the H-H-h clade domains, the catalytic centre comprises two histidines and one hydrophobic residue supplied by β-strands 1, 2 and 5, respectively. The H-Y-[EDQ] clade domains, including PARPs, are characterized by active site composed of histidine, tyrosine and glutamate/aspartate/glutamine whereas in the R-[ST]-E clade domains, active site triad comprises arginine, a polar residue (serine/threonine) and glutamate (Aravind et al., 2015). Three families of the ART clan, PARP, PTS_2-RNA and ART, are present in humans (Table 1), and contain 16, 1 and 4 representatives, respectively. The NEURL-4 ART-like domain family (De Souza & Aravind, 2012), not included in Pfam database, is the fourth human ART-like family. The full catalogue of ADP-ribosylation enzymes is likely far from completion, as one may expect from recent discoveries of novel ART domains in effectors from pathogenic bacteria that perform non-canonical ubiquitination (Akturk et al., 2018; Kalayil et al., 2018; Kim et al., 2018), novel ART/macro pairs in bacterial toxin/antitoxin systems (Jankevicius et al., 2016) and novel human macro domains (Dudkiewicz & Pawłowski, 2019). Other examples of novel ADP-ribosylation players are provided by the viral macro domains, present in many dangerous viruses, including the SARS and SARS-CoV-2 coronaviruses (Saikatendu et al., 2005), and by the recently characterized novel ART-like domain (DUF3715) in the human TASOR protein involved in gene silencing (Douse et al., 2020; Tchasovnikarova et al., 2015).

In this paper, building up on experience in identification of novel enzyme families (Dudkiewicz, Lenart & Pawłowski, 2013; Dudkiewicz & Pawłowski, 2019; Dudkiewicz et al., 2012; Pawłowski et al., 2006; Sreelatha et al., 2018), we identified structural similarity between ART-like catalytic domains and a family of eukaryotic uncharacterized protein domains present in homologs of the leucine-rich repeat containing protein 9 (LRRC9). For clarity, we will call this domain LRRC9-ART. Human LRRC9 is annotated in the UniProt database as protein of unknown function with experimental evidence at transcript level. LRRC9 locus is located on chromosome 14 (q23.1). Gene and protein names refer to the twenty-two leucine-rich repeats located between positions 97 and 246 and 717-1365. According to The Human Protein Atlas (Uhlen et al., 2015), expression of LRRC9 mRNA is enhanced in brain, pituitary gland and testis.

We investigated sequence similarities between the novel and the known ADP-ribosyltransferases. We reconstructed phylogenetic relationships between sequences within LRRC9 domain family and other ART clan families. We also explored sequence variability in the novel ART-like domain in the context of predicted three-dimensional structures and proposed their likely biological functions.

## Materials and Methods

The FFAS03 (Xu et al., 2014), HHpred (Zimmermann et al., 2017) and Phyre2 (Kelley et al., 2015) servers were used to determine distant sequence similarities of LRRC9 central domain to proteins of known structures from public databases. Standard parameters and significance thresholds were selected.

The representative set of LRRC9 ART-like domain sequences was collected by submitting the ART-like domain of the human LRRC9 protein (UniProtKB: Q6ZRR7.2, positions 389-628) to two iterations of JackHMMER (also with standard parameters) ran on the Reference Proteomes database.

The MAFFT program (Katoh, Rozewicki & Yamada, 2019) was used to build the multiple sequence alignments of the novel domains and PARP catalytic domains obtained from the rp75 set from the Pfam database (El-Gebali et al., 2019). In-house scripts were used to merge family-wise multiple sequence alignments according to a FFAS pairwise alignment of representatives of the two families. The WebLogo server (Crooks et al., 2004) was used to visualize results as sequence logos.

The CLANS (Frickey & Lupas, 2004) algorithm was used to visualize close and distant similarities between the putative and the known ADP-ribosyltransferase domains. Sequence similarities up to BLAST *E*-value of 1, 1e^−2^ or 1e^−4^ and the BLOSUM45 substitution matrix were used. In order to acquire collection of the known ADP-ribosyltransferase domains, the following sets of sequences were obtained from Pfam database: ADPrib_exo_Tox rp15, ADPRTs_Tse2 rp15, Anthrax-tox_M rp75, Arr-ms rp15, ART rp15, ART-PolyVal rp15, AvrPphF-ORF-2 rp15, Diphtheria_C rp75, Dot_icm_IcmQ rp35, DUF2441 rp15, DUF952 rp15, Enterotoxin_a rp15, Exotox-A_cataly rp75, NADase_NGA rp35, PARP rp15, Pertussis_S1 rp15, PTS_2-RNA rp15, RolB_RolC rp55 and TNT rp15. Next, collected data were supplemented with three sequence sets representing the following ART-like domain families not included in Pfam database: ESPJ, NEURL4 and DUF3715/TASOR. Sets of ESPJ and SidE domain sequences were obtained by submitting sequences from UniProtKB and Protein NCBI databases (UniProtKB: W0AL45, positions 52-214 and refseq ID: YP_094288.1, positions 686-912, respectively) to two iterations of JackHMMER with standard parameters, ran on UniProtKB database. The input sequence of NEURL4 (UniProtKB: F2TYZ7, positions 102-275) was subject to the same procedure, but extended to three iterations and ran on the Reference Proteomes database. Set of DUF3715 sequences was obtained by submitting the TASOR ART domain (UniProtKB: Q9UK61.3, positions 92-337) to two iterations of JackHMMER with standard parameters. Next, the whole collection of putative and known domain sequences were prepared for the CLANS procedure by clustering with CD-HIT and selecting representatives at a 70% sequence identity threshold (Huang et al., 2010).

In order to determine phylogenetic spread of the novel ART domains, a set of LRRC9 ART-like domain homologs was obtained using JackHMMER search seeded with input sequence UniProtKB: Q6ZRR7.2, positions 389-628. Then, Phylogeny.fr platform (Dereeper et al., 2008) was utilized to construct phylogenetic tree. A multiple sequence alignment was generated using the MUSCLE program (Edgar, 2004). Next, advanced mode of the platform was utilized to build phylogenetic tree using the maximum likelihood PhyML program (Guindon et al., 2009) with standard settings (WAG amino-acid substitution model, four substitution rate categories, aLRT test for bootstrap support). Phylogenetic relationships between homologs were visualized as a dendrogram using the iTOL server (Letunic & Bork, 2016).

Three three-dimensional structure models for the human LRRC9-ART domain were built using homology modelling method Modeller9v21 (Webb & Sali, 2017), I-Tasser server (Yang et al., 2015) and Robetta server (Hiranuma et al., 2020). Simple homology model (Modeller) was based on human PARP10 structure as single template (PDB ID: 3HKV, identity 16%). The I-Tasser multi template model (Yang et al., 2015) were based on best PDB hits identified automatically by the server, top three of them were human tankyrase 2 (PDB ID:3MHK, 3KR7 identity 17%), PARP10 (PDB ID 3HKV, identity 16%) and human poly(ADP-ribose) polymerase 14 (PDB ID 3SMJ, identity 16%). The Robetta model was constructed using a deep learning-based method, trRosetta (Yang et al., 2020).

Putative NAD binding pockets in all modelled structures and chosen native PARP structures were first found and described using DoGSiteScorer fully automatic algorithm for pocket and druggability prediction available at ProteinsPlus server (Volkamer et al., 2012) and independently identified using the COFACTOR algorithm (Roy & Zhang, 2012), an option delivered by the I-Tasser (Iterative Threading ASSEmbly Refinement) server for protein structure and function prediction (Yang et al., 2015) based on threading the query structural model through the BioLiP protein function database. COFACTOR identifies potential functional sites by local and global structure matches and suggests annotations. Putative poses of NAD ligand in the LRRC9-ART trRosetta model were predicted by docking using AutodockVina module in UCSF Chimera (Trott & Olson, 2010). Structures were visualized and analyzed using UCSF Chimera tools (Huang et al., 2014).

The three-domain model for full-length human LRRC9 protein was built using AIDA (Ab Initio Domain Assembly Server) (Xu et al., 2015) based on structures modelled using Modeller9v21 separately for three identified domains: N-terminal LRR region with helical fragment (PDB code of modelling template: 3OJA, 15% identity), ART-like domain (template: 3HKV), and C- terminal LRR region domain (template: 4LSX, 21% identity).

Conservation values of sequence positions in multiple sequence alignment created for the human LRRC9-ART domain homologues (for mapping onto the modelled structures), were derived from Jalview alignment editor (Waterhouse et al., 2009) and were automatically calculated as the number of conserved physicochemical properties for each column of the alignment (Livingstone & Barton, 1993).

The LRR repeats were identified using the LRRFinder tool (Offord, Coffey & Werling, 2010). Gene Ontology analysis of human genes encoding LRR-containing proteins (i.e., their cellular/extracellular localization, molecular function and association with biological processes) was performed with the use of Panther Classification System (Mi et al., 2017). The domain organization of LRRC9 proteins was visualised using the DOG2.0 tool (Ren et al., 2009).

For analysis of protein and mRNA expression, the Proteomics-db, PAXdb, neXtProt and Protein Atlas databases were used (Samaras et al., 2020; Thul & Lindskog, 2018; Wang et al., 2015; Zahn-Zabal et al., 2020). The BioGRID Interaction Database was used to obtain information about possible place of LRRC9 in human protein-protein interaction networks (Oughtred et al., 2019). For prediction of the subcellular localization of LRRC9, the DeepLoc server was used (Almagro Armenteros et al., 2017).

## Results

### Assignment of the central domain of LRRC9 to the ADP-ribosyl clan

**Figure 1:**
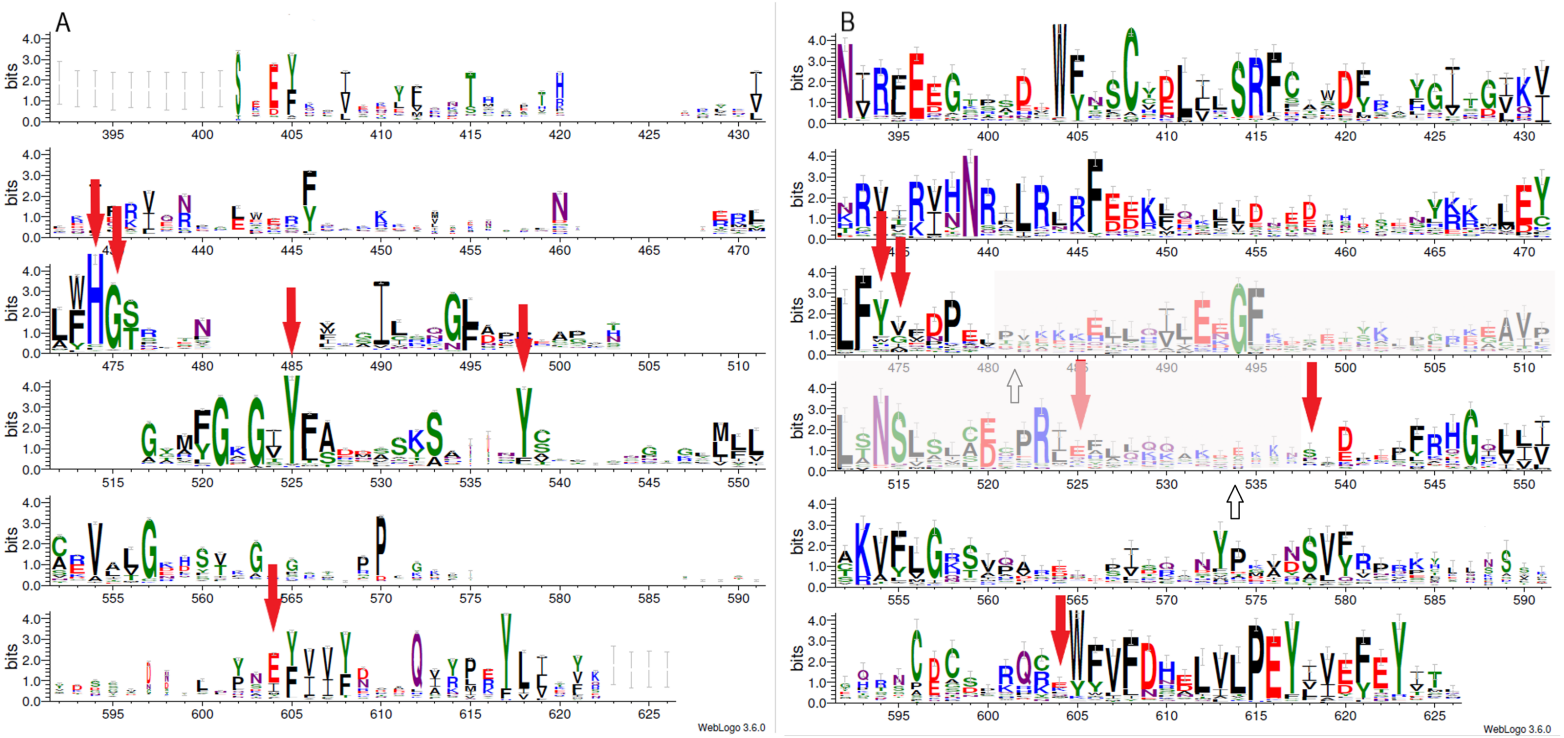
Sequence variability represented as sequence logos for PARP family (A) and LRRC9 ART-like domains (B). Logos were created from two separate MAFFT alignments for PARP family and LRRC9 sequences using WebLogo3 server. The two alignments were merged based on a FFAS03D alignment obtained for human PARP10 and LRRC9-ART domains. Both alignments were trimmed by removing positions with gaps in the human LRRC9 ART domain. Sequence numbering is according to human LRRC9. The catalytically important PARP residues His, Gly, Tyr, Tyr and Glu [matched to positions 474, 475, 525, 538 and 604, respectively, of the human LRRC9 protein] marked with red arrows. Region where FFAS and HHpred alignments between PARP and LRRC9 are inconsistent (between two white arrows) has been faded.

**Figure 2:**
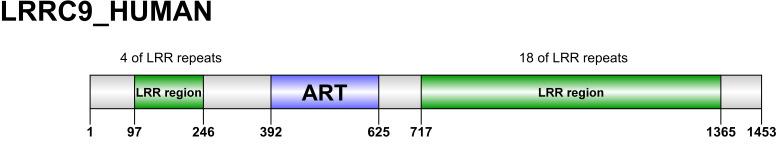
Domain organization of LRRC9 proteins. Sequence numbering as per human LRRC9.

A sequence similarity screen of human proteins for remote homologs of ADP-ribosyltransferases (ARTs) using the FFAS03 method indicated that weak ART similarity might exist in the central region of the LRRC9 protein (Figs. 1, 2). In order to examine in detail this similarity, we used FFAS03, HHpred and Phyre2 servers. All of them are dedicated to protein structure prediction, allowing detection of non-obvious sequence similarity between very distant protein homologs. These three independent bioinformatics tools revealed statistically significant albeit distant sequence similarity of a region of human LRRC9 protein to representatives of the PARP catalytic domain family between positions 392 and 625 (Table 2). Such remote sequence similarity cannot be the basis for drawing a definite conclusion about an enzymatic function of the putative ART domain.

**Table 2:** Structure predictions for the LRRC9 ART-like domain. Structure and distant sequence similarity predictions for human LRRC9 ART-like region (residues 389-628) obtained using different bioinformatics tools. For each method, only the first hit shown.

Bioinformatic tool for structure prediction	Top hit: PDB code, name	Statistical significance for top hit	Region of query aligned to the hit	Sequence identity
FFAS03	**4DVI** Tankyrase 1 with IWR2	Z-score =-12.500	427-625	20%
HHpred	**5LX6,** Human PARP10 (ARTD10), catalytic fragment in complex with PARP inhibitor Veliparib [*Homo sapiens* ]	*E*-value =6.2e−23	392-624	15%
Phyre2	**3HKV**, Human poly(ADP-ribose) polymerase 10, catalytic fragment in complex with an inhibitor 3-aminobenzamide; chain A	Confidence= 100%	395-624	16%

**Figure 3:**
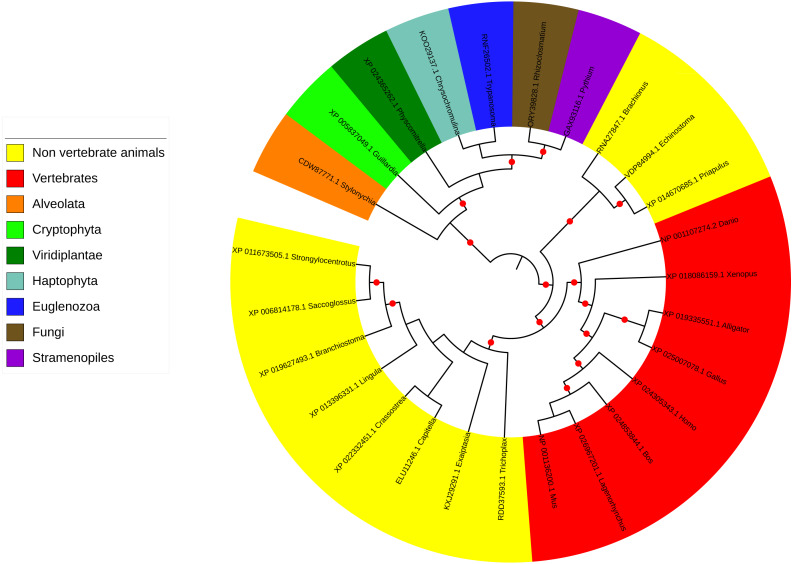
Phylogenetic tree and taxonomic spread of LRRC9 ART-like domains. A maximum likelihood phylogenetic tree for representative LRRC9 ART-like domains. NCBI protein identifiers and genus names shown. Colored by taxon. Red circles denote branches with bootstrap support of at least 75%.

**Figure 4:**
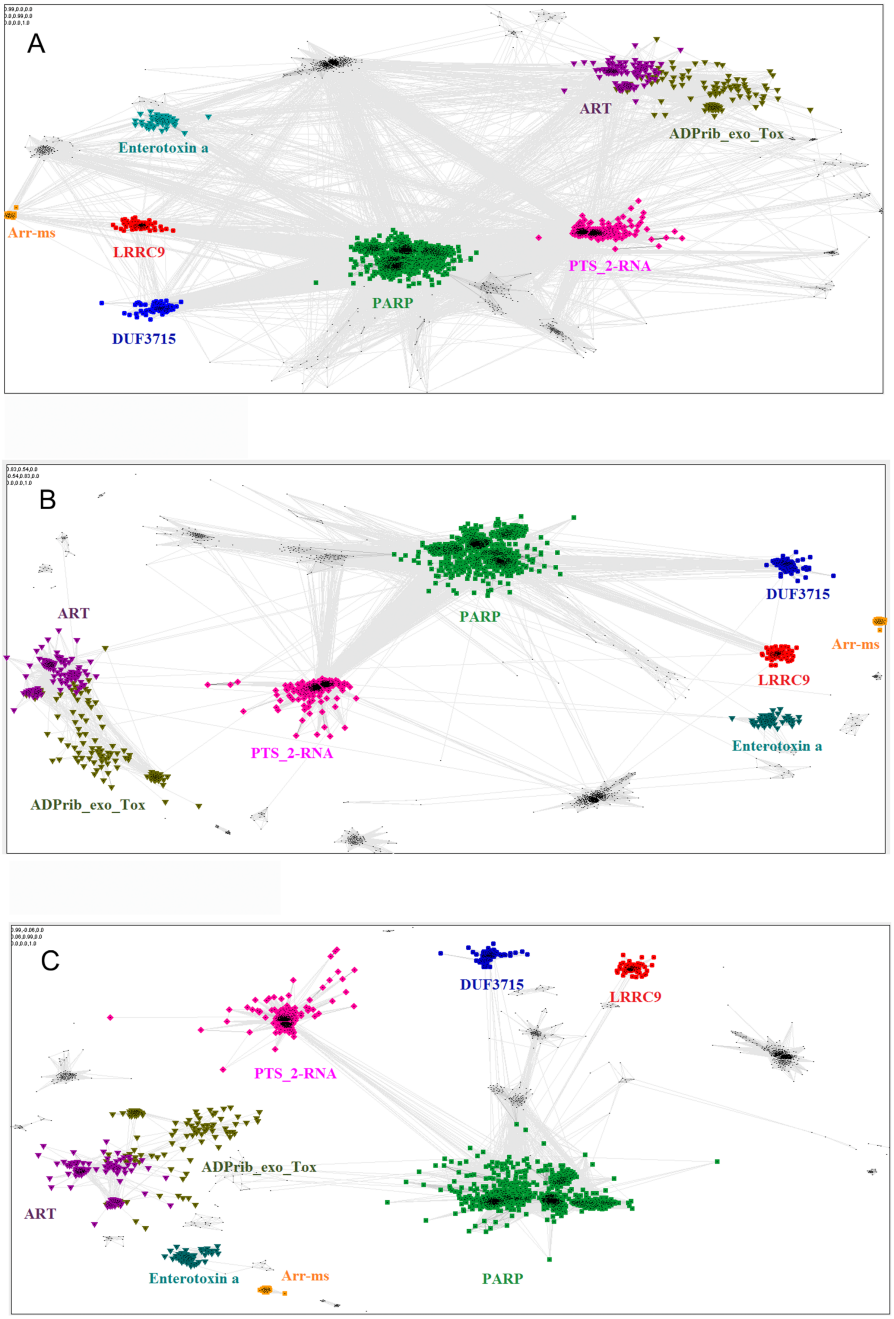
Close and distant sequence similarities between the putative and the known ADP-ribosyltransferase domains, visualized using CLANS algorithm. The graph groups sequences (graph nodes) according to BLAST-derived similarities (edges). (A) Sequence similarities up to BLAST *E*-value of 1, i.e., including distant, non-significant sequence similarities. (B) Up to *E*-value 1e-2. (C) up to *E*-value 1e-4.

The relationship of the novel LRRC9 ART-like family, found in many eukaryotic lineages (Fig. 3), to the known ART families can be visualized using the sequence-based CLANS clustering approach (Fig. 4). CLANS analysis was performed at three different *E*-value levels for BLAST hits for all known families containing ADP-ribosyltransferase domains. In agreement with the FFAS03, HHpred and Phyre results, CLANS results suggest a closer relation between the novel ART family and the PARP domains, noticeable at *E*-value threshold 1, 0.01 and even at 0.0001. This, together with sequence conservation analysis allowed us to hypothesize that LRRC9-ART belongs to the H-Y-[EDQ] clade. The CLANS clustering analysis also suggests that LRRC9-ART should be regarded as a separate family within the ART-like clan/superfamily. The same applies to the DUF3715 family that was recently shown to possess an ART-like structure (Douse et al., 2020). Representatives of both families clearly cluster away from other ART-like clan members and the two novel families are also away from each other. Notably, detection of very distant sequence similarities depends on accurate domain boundary prediction. This may have hindered the recognition of the ART-like domain in TASOR whereas the domain of unknown function DUF3715 as defined in the Pfam database was missing approx. 50 residues at its N-terminus.

### Putative active site of the novel ART-like family

Catalysis of poly-ADP-ribosylation performed by the members H-Y-[EDQ] clade (including PARP domain family) involves three non-consecutive conserved amino acid residues: His, Tyr and an acidic one (Glu or Asp) that can also be substituted by Gln. For consistency, these motifs will be called motifs I, II and III, respectively. Histidine, occurring in the conserved motif I Hx[ST] (Aravind et al., 2015) within β-strand 1, is responsible for binding the 2-OH group of the adenosine ribose of NAD^+^ and NH_2_ group of the nicotinamide amide *via* hydrogen bonds (Cohen & Chang, 2018). Tyrosine (motif II), localized within β-strand 2 (Aravind et al., 2015), interacts with nicotinamide moiety whereas glutamate of β-strand 5 (first position in [QE]x[QED] motif according to Aravind 2018) seems to play a role in maintaining stability of the furanosyl oxocarbenium intermediate (Cohen & Chang, 2018).

Multiple sequence alignment comparison allowed us to identify few similarities and substantial differences between sequences of PARP catalytic domains and ART-like domains, which we identified in human LRRC9 protein sequence in its central part, from position 392 to 625. The tyrosine residue (motif II), one of the catalytic triad elements in PARP, was found to be replaced by a weakly conserved Glu (E525) in human LRRC9-ART. Also, His and Ser residues (from the motif I, His-x-Ser, positions 474–476 in the logo in Fig. 1), highly conserved among PARP enzymatic domain members, were not conserved in LRRC9. The His residue was most often substituted by Tyr. Glutamate, the third element of the catalytic triad (motif III, position 604 in the sequence logo), was replaced by a poorly conserved Lys (Fig. 1). In PARP1, PARP2, PARP10, PARP12 and PARP15 sequences, there is also another important Tyr residue (Tyr932 in human PARP10) that is responsible for nicotinamide stacking (Karlberg et al., 2015). Some authors (Karlberg et al., 2015) include this second Tyr in the group of conserved residues being the hallmark of PARP ART-domains (Karlberg et al., 2015), hence we will use the term “catalytic tetrad HYYE”, representing the non-contiguous catalytic motif H-x(n1)-Y-x(n2)-Y-x(n3)-E. This tyrosine is conserved in PARP (at the logo position 538, Fig. 1A) and not conserved in the LRRC9 ART-like domain (Fig. 1B). Lack of most of the catalytic amino acid residues, evolutionarily conserved in PARP catalytic domains, suggests that LRRC9 ART-like domains may be pseudoenzymes. Interestingly, the FFAS03 sequence alignment between human LRRC9 ART-like and PARP10 ART domains, especially in its central part, is not unequivocal. We obtained different alignments using FFAS03 and HHpred servers and even in FFAS results, there are some suboptimal alignment paths to be considered (Figs. S1 and S2). According to the FFAS alignment, PARP region flanked by two tyrosines belonging to the catalytic tetrad HYYE is aligned to a poorly conserved region 525–538 of the LRRC9 ART-like domain (Fig. 1A), but three positions upstream and downstream of this fragment there are two distinct motifs of LRRC9-ART domain: PR[IL] and [DE]xxxFRHG, respectively. This suggests that sequence-based alignments may be inaccurate in this region and one of both above-mentioned LRRC9 conserved motifs may in fact be involved in the active site. Also, the strongly conserved D-(5)-PEY-(2)-EFEY motif in LRRC9 (logo positions 609-623), although not aligned to PARP active site, may be speculated to be functionally important.

**Figure 5:**
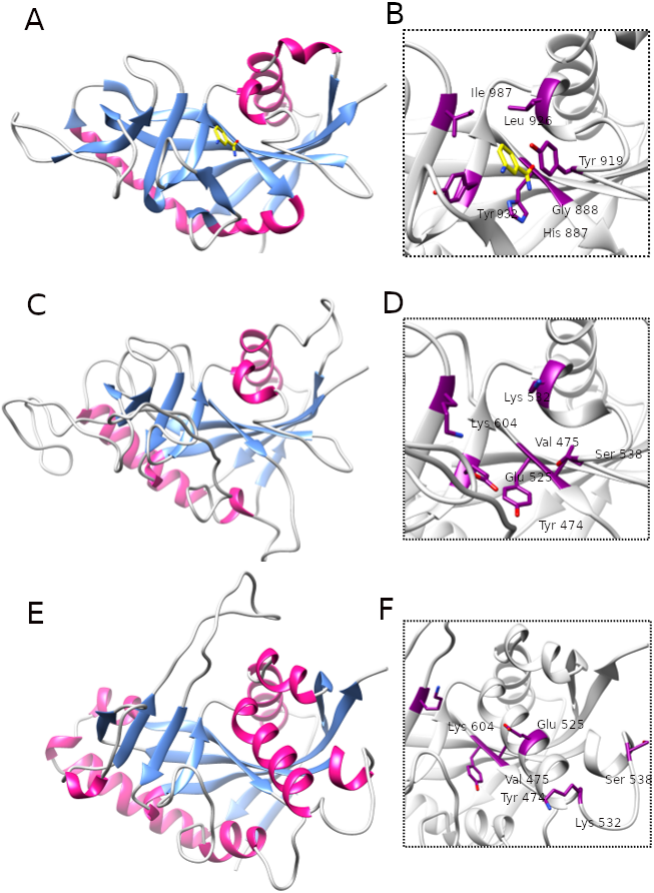
Comparison of ART-like domains and their active site regions. Overall structures of ART domains (A, C, E) and close-up active site regions (B, D, F) of: PARP10, PDB ID: 3HKV (A, B), human LRRC9-ART modelled on the PARP10 template (C, D), and human LRRC9-ART structure modelled using TrRosetta (E, F). Catalytic PARP residues and their counterparts in the modelled structures are depicted as sticks. The ligand shown in panels (A, B) is 3-aminobenzamide, a PARP inhibitor (yellow). Coloring by secondary structures.

### Structural analysis of the homology model

Because distant homology detection methods did not offer us consensus for template selection, we decided to create model based on three different approaches: classical one-template homology modeling (Modeller), reassembling structural fragments from threading templates using Monte Carlo simulations (I-Tasser approach, best model with C-score 0.5) and deep learning based methods which use covariance signals obtained from sequence alignments to predict inter-residue distances (trRosetta, best model confidence: 0.70). In the next step, we used DogSiteScorer to analyse the structure models to identify putative ligand binding pockets and compare them to the pockets in known PARP structures (Table S1, Fig. S3) To illustrate uncertainty of structural arrangements of putative ligand-binding residues identified by FFAS03 alignments, we used the homology model (PARP10/3HKV template-based) and the trRosetta (deep learning) model which according to ligand pocket analysis are more likely to accommodate a ligand. Comparison of the template 3HKV structure and these two alternative models is presented in Fig. 5. To answer the question if the observed distant sequence similarity between LRRC9-ART and PARP families can translate into functional similarity, we focused first on the known structural determinants of ADP-ribosyltransferase activity (Karlberg et al., 2015) and relating these to LRRC9-ART features. In classical PARPs, like PARP1 and PARP2, possessing a poly-ADP-ribosylation (PAR) activity, besides the canonical H-x(n1)-Y-x(n2)-Y-x(n3)-[EI] tetrad mentioned above, there are two other key positions associated with structural and functional roles of these proteins: glycine residue with nicotinamide anchoring function and an additional catalytic amino acid, lysine 903 (according to PARP1 numbering) (Karlberg et al., 2015). In PARP10 and PARP12 with mono-ADP-ribosylation (MAR) activity, instead of this catalytic lysine, there is a leucine or tyrosine residue. Investigating profile-profile alignments for human LRRC9 and PARP10 ART domains one can easily notice that only the first of the canonical tetrad residues is conserved, although substituted (H->Y). Thus, histidine and glycine responsible for nicotinamide anchoring and binding are replaced by tyrosine and valine (Figs. 5B, 5C). In PARPs, the glycine makes two conserved H-bonds to the nicotinamide via main chain carbonyl oxygen and amine hydrogen. These should not be disrupted by valine substitution as the side chain points outward from the active site. Interestingly, in LRRC9-ART at position 532 there is a weakly conserved Lys, as in PARP family members with poly-ADP-ribosylation (PAR) activity. However, instead of a second Tyr (logo position 538) and Glu/Ile residues (604), there are poorly conserved Ser and Lys.

To identify possible ligand pockets in modelled structures we used DoGSiteScorer. For comparison, we also analysed classic PARP structures where ligand binding sites are known and described. We selected PARP1 (ART domain with poly-ADP-ribosylation activity), PARP10 (mono-ADP-ribosylation activity) and two examples of inactive PARP/ART-like domains: PARP13 and TASOR. In case of native structures, DoGSiteScorer correctly identified pockets in the vicinity of PARP hallmark H-Y-Y-E tetrad or its counterpart as the best scoring ones. In case of models, pockets detected in the neighborhood of H-Y-Y-E aligned residues were the best scoring ones for I-Tasser and trRosetta models (Table S1). Overall shape, volume and surface of modelled pockets were very different, but the best score (close to the score obtained for native NAD binding pocket in PARP1) and parameters mostly resembling active PARPs were identified in trRosetta model of LRRC9-ART (Fig. S3). Thus, this model appears to best capture the putative ligand binding site in LRRC9.

Additionally, to investigate possible ligands for the LRRC9-ART domain we used COFACTOR. COFACTOR is a functional annotation tool based on threading of the protein structure query through a database of ligand-protein binding interactions by local and global structure matches to identify functional sites (Zhang, Freddolino & Zhang, 2017). COFACTOR suggests the best candidates fitting into the ligand binding site of a given structure. Adenosine monophosphate (AMP) turned out to be among the best scoring ligands for the LRRC9-ART homology model while NAD, a typical ligand for ART domains, was absent from the predicted ligand list. AMP molecule, which differs from NAD by the lack of nicotinamide mononucleotide moiety, is smaller and probably fits better in the modelled LRRC9-ART ligand binding groove. The docked AMP molecule pose was deduced from homology to crystal structure of the catalytic domain of *Pseudomonas aeruginosa* exotoxin A (PDB ID: 1DMA) which catalyzes the transfer of ADP ribose from NAD to elongation factor-2 in eukaryotic cells and hence inhibits protein synthesis. These docking results do not suggest that AMP is a physiological ligand of LRRC9-ART, rather, they provide a likely binding pose of the AMP moiety of NAD. For trRosetta model, COFACTOR found tankyrase-1 complexed with a PARP inhibitor, P4L, as the best ligand binding site analogue. Here, NAD molecule was not present on the list of ligands complexed with identified binding site structural homologues.

To examine the possibility of NAD binding, we attempted to dock the full NAD molecule into the LRRC9-ART hypothetical active site in the trRosetta (deep learning) structure model. The ligand was constructed in UCSF Chimera build tool and then it was docked using Autodock Vina procedure without specific constraints to the binding groove identified by DoGSiteScorer (Fig. S4B). The docking procedure located NAD in the best scoring pocket in few different poses (Fig. S4A), the best one with a good docking score of −6.8 (Fig. S4C). This suggests we cannot exclude the LRRC9-ART domain, at least as modelled using trRosetta approach, can bind NAD. However, docking a ligand to a structure model built using very distant templates has to be seen as speculative.

Replacing the catalytic Glu residue by Ile is characteristic for PARP family members having mono-ADP-ribosylation (MAR) activity (Karlberg et al., 2015). Among FFAS/HHpred hits for LRRC9-ART domain, proteins with MAR, as well as PAR activity and with no confirmed ADP-ribosylation activity appeared. In the sequence logos of ART and LRRC9-ART, there are no common conserved motifs between the two domain families. In LRRC9, the second tyrosine of the HYYE tetrad is replaced by glutamine 525, which is noticeably less conserved. Another highly conserved motif of the PARP family, YxEY[VI][VI][FY], containing catalytic Glu of the HYYE tetrad does not align to a relatively similar motif visible in the LRRC9-ART domain EY[VI][VI]E[FY]EY (logo positions 616-623). As mentioned earlier, these motifs may correspond to each other, and the apparent misalignment may be just an artifact.

Figure S4 shows the trRosetta model of LRRC9-ART structure, with NAD molecule docked in the predicted active site. Figure S4B focuses on the same residues as Fig. 5 but colored according to conservation in the LRRC9-ART family. Remarkably, only tyrosine 474 and valine 475 (out of the residues aligned to the catalytic tetrad) are quite strongly conserved in the whole family. The rest of LRRC9 amino acids aligned to the classical HYYE tetrad residues in the ligand-binding hydrophobic pocket are not highly conserved. This approach of docking a ligand to a structure model built using remote sequence similarity cannot be regarded as authoritative; it is only preliminary and can give a general idea of ligand placement, but no detailed information on its interactions. The model presented in surface representation (Fig. S4C) shows the conserved residues are grouped at the bottom of the putative ligand binding groove.

The ART-like domain described here is only one region of approx. 200 residues within the whole LRRC9 protein molecule, which is 1453 amino acids long, with clear similarity to leucine-rich repeat-containing domains in its N-and C-terminal parts, and a very long helical “spine” in the center. We were tempted to construct the full-length molecule model, based on homology to LRR-containing templates and using the already modelled ART-like domain structure. The results are shown in Fig. S5. In the full-length structure model proposed here, a large groove is surrounded by LRR “horseshoes” from three sides and an ART-like domain from the fourth. The size of the groove seems to suggest a possibility of binding a large molecule, e.g., a protein rather than a small cofactor. It is tempting to speculate that LRRC9, binding some “bait”, rearranges its conformation and activates the enzymatic ART-like domain, thereby triggering a cellular signal. However, the full-length model is only an illustration of possible domain arrangements.

### Taxonomic distribution of the LRRC9-like proteins

The LRRC9 protein is widespread in several of the major eukaryotic lineages. It is found in *Alveolata* and *Stramenopiles* (from the TSAR supergroup Burki et al., 2020)*, Cryptophyta*, *Haptophyta, Viridiplantae, Fungi and Metazoa (Opisthokonta*) and *Euglenozoa* while absent from several other main lineages, e.g., *Hemimastigophora*, CRuMS, *Metamonada*, *Malawimonadida*, *Ancyromonadida*, *Ancoracysta*, *Picozoa* (Fig. 3). Thus, this protein appears to be an ancestral eukaryotic feature, that was subject to losses in a number of lineages.

ART-like domain is present in all vertebrate classes as well as in 11 non-vertebrate animal phyla (*e.g., Cnidaria, Echinodermata, Brachiopoda, Mollusca, Annelida, Hemichordata, Chordata*). However, notable is its absence in several animal phyla, some of which include important model organisms (e.g., *Arthropoda*, *Nematoda*, *Porifera*; see Fig. 3). Also, strikingly, although LRRC9-ART domains are found in some *Viridiplantae*, e.g., mosses, green algae, liverworts, and club mosses, they are absent from flowering plants with the exception of the water lily *Nymphaea colorata*. Notably, water lilies belong to *Magnoliophyta*, a taxon thought to have diverged earliest from the lineage leading to most extant flowering plants (Zhang et al., 2020). Altogether, this taxonomic spread suggests an ancient function for LRRC9, common to many diverse eukaryotes.

## Discussion

Because the regions of LRRC9-ART domain corresponding to ADP-ribosyltransferase active sites are poorly conserved, the most straightforward conclusion appears to be that LRRC9-ART is a pseudoenzymatic domain. However, as exemplified by the recent cases of two pseudokinases identified by us, SelO and SidJ, apparent pseudoenzymes may in fact perform enzymatic functions somewhat similar to those performed by their “non-pseudo” cousins (Black et al., 2019; Sreelatha et al., 2018). Also, many inactive pseudoenzymes engage their non-pseudo cousins and modulate their activity, e.g., allosterically (Adrain, 2020). Thus, we discuss the significance of the LRR repeats flanking the ART-like domain while speculating that the central domain either performs by itself an ADP-ribosylation-related catalytic function, or modulates such function performed by yet another molecule.

Apart from the ART-like domain, LRRC9 proteins comprise a variable number of leucine-rich repeats (LRRs) flanking the ART domain (Fig. 2). In all the homologs analysed, the LRR-ART-LRR domain architecture is conserved in evolution. The N-terminal region possesses 1-6 LRR repeats while the C-terminal region possesses 11-20 such repeats. The N-terminal repeats and C-terminal repeats are most similar to the LRR repeats from proteins such as Leucine Rich Repeat Containing 23 (LRC23) and TLR4 Interactor with leucine-rich Repeats (TRIL), respectively, however, degree of similarity between LRR motifs from different proteins is generally low and makes it difficult to draw specific functional conclusions. The LRR motifs typically have a length of about 20-30 amino acid residues and are rich in leucines or other aliphatic residues (typically seven per motif, localized at positions 2, 5, 7, 12, 16, 21 and 24 within the consensus LRR sequence) (Kobe & Deisenhofer, 1994). The LRR motifs may form various combinations of two or three secondary structures, usually involving two stretches of a β-strand, e.g., two β-strands and an α-helix. The LRR repeats typically fold into a horseshoe-like conformation composed of helices present on its convex face and parallel β-sheet localized on the concave one (Enkhbayar et al., 2004). LRR motifs determine functionality of LRR-containing proteins, enabling them to make protein-protein interactions (Kobe & Deisenhofer, 1994) as well as to bind various target structures such as small molecule hormones, lipids and nucleic acids (Helft et al., 2011). The non-globular shape of LRR motifs may facilitate the contact between LRR-containing proteins and the target structures, increasing the interaction area (Kobe & Deisenhofer, 1994). For example, ribonuclease inhibitor is able to bind pancreatic ribonucleases and inhibit their enzymatic activity. This interaction may affect RNA turnover in the context of angiogenesis (Kobe & Deisenhofer, 1993).

Employing the LRR motifs, LRR proteins perform various functions. They may play a role in signal transduction and cell development as well as they are able to participate in RNA splicing and effective response to DNA damage (Kobe & Deisenhofer, 1995). Many LRR-containing proteins are adhesive molecules that perform important functions in processes such as regulation of collagen-fibril formation, osteogenesis, myelination and platelet adhesion at a site of vascular injury. Many proteins with LRR motifs play a role in signal transduction as specific receptors. For example, in response to LPS stimulation, LRR-containing CD14 protein induces tyrosine phosphorylation of intracellular proteins to stimulate antibacterial activity of macrophages. Other receptors exhibit specificity to gonadotrophins such as luteinizing hormone, chorionic gonadotrophin and follicle-stimulating hormone (Bluhm et al., 2004).

The LRR domains, structurally conserved in evolution, occur widely within plant, invertebrate and vertebrate proteins responsible for innate immunity. Due to their ability to mediate protein-protein interactions, LRR repeats determine signaling function of many pattern recognition receptors (PRRs), such as Toll-like receptors (TLRs) and NOD-like receptors (NLRs), by conditioning their affinity to various ligands, e.g., viral, bacterial, fungal and parasite antigens (Ng & Xavier, 2011; Ng et al., 2011). On the other hand, ADP-ribosylation is known to be involved in regulation of the host immune response (Fehr et al., 2020; Rosado et al., 2013). It may direct intracellular signaling to parthanatos (type of programmed death) (Fatokun, Dawson & Dawson, 2014; Greenwald & Pierce, 2019; Hoch & Polo, 2019), whereas such scenario implies induction of inflammatory response to infection. Poly-ADP-ribosylation is known to influence the stability of transcripts encoding proinflammatory cytokines (Ke et al., 2019). PARP-dependent chromatin modification may counteract progression of viral infection. ADP-ribosylation also influences other innate immune mechanisms such as NF-kB expression, phagocytosis and macrophage polarization (Kunze & Hottiger, 2019). Here, co-occurrence of ART and LRR domains within the LRRC9 protein suggests that they may act together to support a version of host innate immunity. In this context, the LRR domains could perform signal sensors function, and the ART domain might play the effector role.

Overall, in the human proteome there are 234 proteins containing LRR repeats. The molecular functions significantly overrepresented among these proteins include peptide binding (11 proteins, FDR *p*-value 8E-6), ubiquitin-protein transferase activity (11 proteins), heparin binding (7 proteins) and G-protein-coupled peptide receptor activity (7 proteins). However, as many as 182 human LRR proteins are functionally uncharacterized, according to Panther-db. Human proteins containing LRR repeats are involved in processes such as neurogenesis (17 proteins, FDR 1E-5), ubiquitin-dependent protein catabolic process (11 proteins), positive regulation of innate immune response *via* toll-like receptor signaling pathway (10 proteins, FDR 8E-10).

The ART-like domain combined with LRR motifs has precedents. As many as 35 human LRR proteins possess enzymatic domains. Notable here are the NLRP immune sensors/effectors containing NACHT ATP-ase domains, totaling 17 human proteins (Martinon & Tschopp, 2007; Pawlowski et al., 2001). Other enzymes with LRR motifs include protein kinases (11 proteins), nucleases and peroxidases. Eight human LRR proteins also possess F-box motifs and are involved in ubiquitin ligase complexes.

A bioinformatics study analyzing non-LRR regions embedded within LRR proteins identified the LRRC9 family as containing such a “non-LRR island”, noted a few conserved sequence motifs and homologs in a number of non-vertebrate eukaryotes but did not observe similarity to any characterized functional domains (Matsushima et al., 2009).

As no high-quality protein expression data is available for LRRC9, it is annotated as “existence validated on transcript level” in the neXtProt (Zahn-Zabal et al., 2020) database. According to PAXdb (Wang et al., 2015), LRRC9 protein expression is elevated in tonsil, esophagus and seminal vesicle tissues. This may suggest that LRRC9 protein expression is limited to specific circumstances, and is not easily captured in typical tissue proteomics experiments. However, recently, a study aimed at validating the “missing proteins” confirmed the existence of LRRC9 protein in human sperm by applying targeted mass spectrometry and antibody-based methods (Carapito et al., 2017). LRRC9 mRNA expression is highest within the brain as well as endocrine and male reproductive tissues, i.e., pituitary gland and testis, respectively (Protein Atlas database). The apparent discrepancy between mRNA and proteomics expression data for LRRC9 may be related to technical difficulties (e.g., too few unique peptides of sufficient length). According to the DeepLoc prediction, the subcellular localization of LRRC9 is cytoplasmic (likelihood 0.79).

For an uncharacterized protein, its physical interactors may shed light on its function. However, according to the BioGRID database, affinity capture-mass spectrometry analysis provided evidence that LRRC9 physically interacts with a single protein, ZFP36L2 (Noguchi et al., 2018). ZFP36L2 is an RNA-binding protein regulating cell cycle (Galloway et al., 2016) that contributes to pathogenesis of pancreatic ductal adenocarcinoma (PDAC). ZFP36L2 is responsible for an increase in cancer cell aggressiveness (Yonemori et al., 2017). A Kaplan–Meier survival analysis (Protein Atlas) shows that LRRC9 mRNA expression is positively correlated with survival among patients with pancreatic cancer (*p* = 0.0034), although the gene is not classified as prognostic. This may suggest an antagonistic relation between LRRC9 and ZFP36L2 activities in the context of ZFP36L2-dependent promotion of PDAC progression. However, the LRRC9-ZFP36L2 interaction was only reported in a high-throughput experiment, and further investigation is required here.

LRRC9-ART should be classified as a separate ART-like domain family, not only because of the co-occurrence with LRR domains, but primarily because the LRRC9-ART similarity to known ARTs is very remote, and the conservation patterns in LRRC9-ART family are distant from those typical for either PARP or TASOR families.

Pseudoenzymes are characterized by the lack of enzymatic activity despite the common evolutionary origin and structural similarity to catalytically active homologues (Jeffery, 2020; Murphy, Mace & Eyers, 2017; Ribeiro et al., 2019). Pseudoenzymes do occur among PARP family members, including PARP13 that is distinguished by amino acid substitutions and structure changes preventing catalysis. Similarly to LRRC9-ART, PARP13 catalytic domain is characterized by lack of conserved His and Glu residues (Karlberg et al., 2015). It has been shown that substitution of catalytic His with Ala within the ART domain of PARP1 substantially decreases its catalytic activity (Marsischky, Wilson & Collier, 1995). The results were consistent with previous studies on diphtheria toxin, another member of H-Y-[EDQ] clade. Replacement of catalytic His reduced the toxin’s activity by at least 70-fold (Blanke et al., 1994). Thus, the LRRC9-ART family, lacking most of the ART active site, can be hypothesized to be pseudoenzymes. However, one cannot exclude the possibility that this ART-like domain has retained or regained the ART catalytic function, or acquired a novel enzymatic activity, in both cases employing an atypical and/or migrated active site. Such a scenario has been recently reported for apparent pseudokinases, SelO and SidJ, that were shown to be AMPylases and polyglutamylases, respectively (Black et al., 2019; Sreelatha et al., 2018). It can also be envisaged that LRRC9 may require another protein factor to complement its active site, similarly to ADP-ribosyltransferases PARP1 and PARP2 that form complexes with HPF1 which contributes a “missing residue” to the otherwise “incomplete” ART active site (Suskiewicz et al., 2020). Another precedent for a “third party” protein required for ART activity is provided by the PARP9/DTX3L heterodimer whereas the otherwise inactive PARP9 needs the interaction partner to become active (Yang et al., 2017).

On the other side, in eukaryotic cells, poly-ADP-ribosylating proteins may perform some of their functions without utilizing the enzymatic activity. For example, PARP1 participates in NF-kB-dependent gene transcription *via* two different mechanisms but only one requires poly-ADP-ribosylation whereas the second seems not to involve PARP1 enzymatic function and depends on the structure of the protein (Hassa et al., 2003; Weaver & Yang, 2013). Both a pseudo-enzymatic character of LRRC9-ART, and migration of the active site are possible for this intriguing novel family. Experimental studies are needed to confirm one of those hypotheses. The unique, conserved domain architecture of LRRC9, suggests that this mysterious protein family could be involved in a defense mechanism, with some analogies to the innate immune system, coupling within a single molecule the detection of foreign objects (LRRs) and downstream signalling (ART domain).

##  Supplemental Information

10.7717/peerj.11051/supp-1 Supplemental Information 1 Comparison of ligand binding pockets detected in human PARP1, PARP10, PARP13, TASOR ART domains and three models of human LRRC9 ART-like domain Description of best scoring pockets identified by DoGSiteScorer (Volkamer et al., 2012) in four ART domain structures and three modelled LRRC9 ART-like domains obtained using three different methods. In every case the best scoring pocket found in the vicinity of PARP/ART NAD binding site tetrad (or its counterparts in inactive domains or models) was selected for comparison.

10.7717/peerj.11051/supp-2 Supplemental Information 2 Sequence logos for PARP family (A) and LRRC9 homologues (B) matched based on HHpred alignment This is an alternative version of Figs. 1A and 1B, where logos are matched using a FFAS03 alignment.

10.7717/peerj.11051/supp-3 Supplemental Information 3 Suboptimal alignment paths for human LRRC9 and human PARP10 ART-like domains shown by the FFAS03 server Optimal alignment returned by FFAS can also be displayed on the graph as a series of near-diagonal lines (green). An element ( M , N ) of the similarity matrix is a profile–profile similarity score of a position M in the first sequence and a position N in the second sequence. Visualization of this matrix is an M by N heat map with a color scale ranging from blue (the highest similarity between N and M ) to red (the lowest similarity). The presence of regions of high similarity not overlapping with actual alignments (green lines) suggest the presence of a sequence repeat or alternative alignment path.

10.7717/peerj.11051/supp-4 Supplemental Information 4 Comparison of best ligand binding pockets in four native and three modelled ART-like domains (superimposed) Structures and structures models (in the same orientation) shown in ribbon representation, best scoring pockets shown as surfaces (colored according to the scores: rank 1 - yellow, rank 2 - pink). PARP/ART NAD binding tetrad residues or their counterparts are shown in stick representation. Active human ART domains: PARP1 (PDB ID: 6BHV) (A), PARP10 (PDB ID:3HKV) (B). Inactive human ART-like domains: PARP13 (PDB ID: 4X52) (C) and TASOR (PDB ID:6TL1) (D). Human LRRC9 models: homology model with 3HKV template (E), I-Tasser model (F), TrRosetta model (G).

10.7717/peerj.11051/supp-5 Supplemental Information 5 Results of NAD docking to human LRRC9 ART-like domain structure modelled using TrRosetta (A) Ligand poses identified by AutoDock Vina procedure (surface representation) filling the identified ligand binding pocket, see Fig. S3 G. (B) NAD poses in stick representation in the binding grove, model in ribbon representation, colored according to conservation values of sequence positions in sequence alignment of human LRRC9-ART domain homologues. PARP H-Y-Y-E tetrad residues counterparts are depicted in stick representation. (C) Best scoring NAD pose in the putative binding grove, LRRC9-ART structure in surface represenattion. Colored residues: orange - Tyr 474, Val 475, blue - Lys 604, green - Glu 525.

10.7717/peerj.11051/supp-6 Supplemental Information 6 Structure model of the full-length human LRRC9 protein, including LRR domains (A) ribbon representation (B) surface representation (two orientations). Magenta - LRRC9-ART domain, blue - N-terminal LRR region, olive green - C-terminal LRR region, navy blue - helical fragment between ART domain and LRR. Putative ART active site residues are marked light green.

10.7717/peerj.11051/supp-7 Supplemental Information 7 Sequence logos for LRR regions of LRRC9, and domain arrangement Domain organization of human LRRC9 with sequence logos for N-terminal and C-terminal LRR regions.

10.7717/peerj.11051/supp-8 Supplemental Information 8 Multiple sequence alignment for the PARP family. Source dataset for Fig. 1A The alignment is trimmed by removing positions that are aligned to gaps in human LRRC9 in FFAS alignment.

10.7717/peerj.11051/supp-9 Supplemental Information 9 Multiple sequence alignment for human LRRC9 ART-like domain and its homologues. Source dataset for Fig. 1B The alignment is trimmed by deleting positions with gaps in human LRRC9.

10.7717/peerj.11051/supp-10 Supplemental Information 10 CLANS output file for ART-like families, using *E*-value threshold 0.0001. Source file for Fig. 4A

10.7717/peerj.11051/supp-11 Supplemental Information 11 CLANS output file for ART-like families, using *E*-value threshold 0.01. Source file for Fig. 4B

10.7717/peerj.11051/supp-12 Supplemental Information 12 CLANS output file for ART-like families, using *E*-value threshold 1. Source file for Fig. 4C

10.7717/peerj.11051/supp-13 Supplemental Information 13 Multiple sequence alignment file (uncharted) for LRRC9 ART-like domain homologues, source file for phylogenetic tree from Fig. 2

10.7717/peerj.11051/supp-14 Supplemental Information 14 Structure model of human LRRC9 ART-like domain constructed based on PARP10 template (source for Fig. 5B)

10.7717/peerj.11051/supp-15 Supplemental Information 15 Structure model of human LRRC9 ART-like domain constructed on three templates (source for Fig. 5C)

10.7717/peerj.11051/supp-16 Supplemental Information 16 Full length structure model of human LRRC9 - source file for Suppl. Fig. 4 The model includes the central ART-like domain and the LRR regions.

10.7717/peerj.11051/supp-17 Supplemental Information 17 HHM based alignment of C-terminal LRR region of LRRC9 and its structural template The alignment between C-terminal (LRR) fragment of human LRRC9 and sequence of the template structure (PDB: 4LXR). Used to create full-length LRRC9 protein model.

10.7717/peerj.11051/supp-18 Supplemental Information 18 HHM based alignment of N-terminal LRR region of LRRC9 and its structural template The alignment between N-terminal (LRR) fragment of human LRRC9 and sequence of the template structure (PDB: 3OYA). Used for create full-length LRRC9 protein structure model.

10.7717/peerj.11051/supp-19 Supplemental Information 19 FFAS03 based alignment of LRRC9-ART domain and its structural template The alignment between ART-like region of human LRRC9 and sequence of the human PARP10 (PDB: 3HKV). Used for create LRRC9-ART domain structure model and merging/matching MSA for PARP family and MSA for LRRC9 sequences.

## Additional Information and Declarations

### Data Availability

The following information was supplied regarding data availability: Raw data (including protein sequence alignments, CLANS analysis files, subsignificant alignment plots and PDB file for structure model) are available in the Supplemental Files.
