# Supplementary material for: A novel predicted ADP-ribosyltransferase-like family conserved in eukaryotic evolution
Source: PeerJ. 2021 Mar 10;9:e11051. doi: 10.7717/peerj.11051 (PMC7955679; doi:10.7717/peerj.11051)
Supplement: Supplemental Information 1 — Description of best scoring pockets identified by DoGSiteScorer (Volkamer et al., 2012) in four ART domain structures and three modelled LRRC9 ART-like domains obtained using three different methods. In every case the best scoring pocket found in the vicinity of PARP/ART NAD binding site tetrad (or its counterparts in inactive domains or models) was selected for comparison. [file peerj-09-11051-s001.docx]

| **PDB code or model** | 6BHV | 3HKV | 4X52 | 6TL1 | Model 1  (Modeller)  (3HKV based) | | Model 2  ( I-Tasser) | Model 3  (Robetta) |
| --- | --- | --- | --- | --- | --- | --- | --- | --- |
| **Protein name** | **PARP1** | **PARP10** | **PARP13** | **TASOR** | **LRRC9** | | | |
| **ADP-ribosylation activity:** | P (poly-) | M (mono-) | inactive | inactive | not determined | | | |
| NAD binding pocket hallmark residues  (or counterparts of PARP H-Y-Y-E tetrad)  **names of residues not included in the best pocket are italicized* | **H**-H862, G863,S864,  **Y**- Y896,K903, **Y**-Y907,  **E**-E988 | **H**-H887, G888,T889,  **Y**-Y919,L926, **Y**-Y932,  **E**-I987 | **H**-Y787, A788,T789,  **Y**- Y819,Y826, **Y**-C831(N830), **E**-V876 | **H**-l184, M185,V186,  **Y**- Y217,L224, **Y**-L225,  **E**-Q299 | **H**-Y474, V475, F476,  **Y**-E525, K532,  **Y**-S538,  **E**-K604 | ***H****-Y474, V475, F476*,  **Y**-E525(I511?), *K532,*  ***Y****-S538,*  **E**-K604 | | **H**-Y474, V475, F476,  **Y**-E525 (I511?), *K532,*  ***Y****-S538*,  **E**-K604 |
| Parameters of the best pocket identified by DoGSiteScorer in the vicinity of the above hallmark residues | | | | | | | | |
| **simple score** | 0.69 | 0.32 | 0.48 | 0.25 | 0.36 | | 0.31 | 0.56 |
| **rank** | 1 | 1 | 1 | 1 | 2 | | 1 | 1 |
| **Size and shape descriptors** | | | | | | | | |
| volume [Å³] | 1090.88 | 478.14 | 708.93 | 474.94 | 586.05 | | 515.2 | 821.82 |
| surface [Å²] | 1192.78 | 613.62 | 1032.85 | 584.84 | 866.83 | | 690.19 | 1075.79 |
| depth [Å] | 19.69 | 13.28 | 21.08 | 12.82 | 18.44 | | 17.73 | 17.85 |
| **Amino acid composition** | | | | | | | | |
| apolar amino acid ratio | 0.32 | 0.39 | 0.34 | 0.46 | 0.40 | | 0.36 | 0.37 |
| polar amino acid ratio | 0.47 | 0.48 | 0.51 | 0.42 | 0.20 | | 0.39 | 0.32 |
| positive amino acid ratio | 0.18 | 0.13 | 0.07 | 0.08 | 0.20 | | 0.21 | 0.24 |
| negative amino acid ratio | 0.03 | 0.00 | 0.07 | 0.04 | 0.20 | | 0.04 | 0.08 |
