# Supplementary figures and images for: A novel predicted ADP-ribosyltransferase-like family conserved in eukaryotic evolution
Source: PeerJ. 2021 Mar 10;9:e11051. doi: 10.7717/peerj.11051 (PMC7955679; doi:10.7717/peerj.11051)

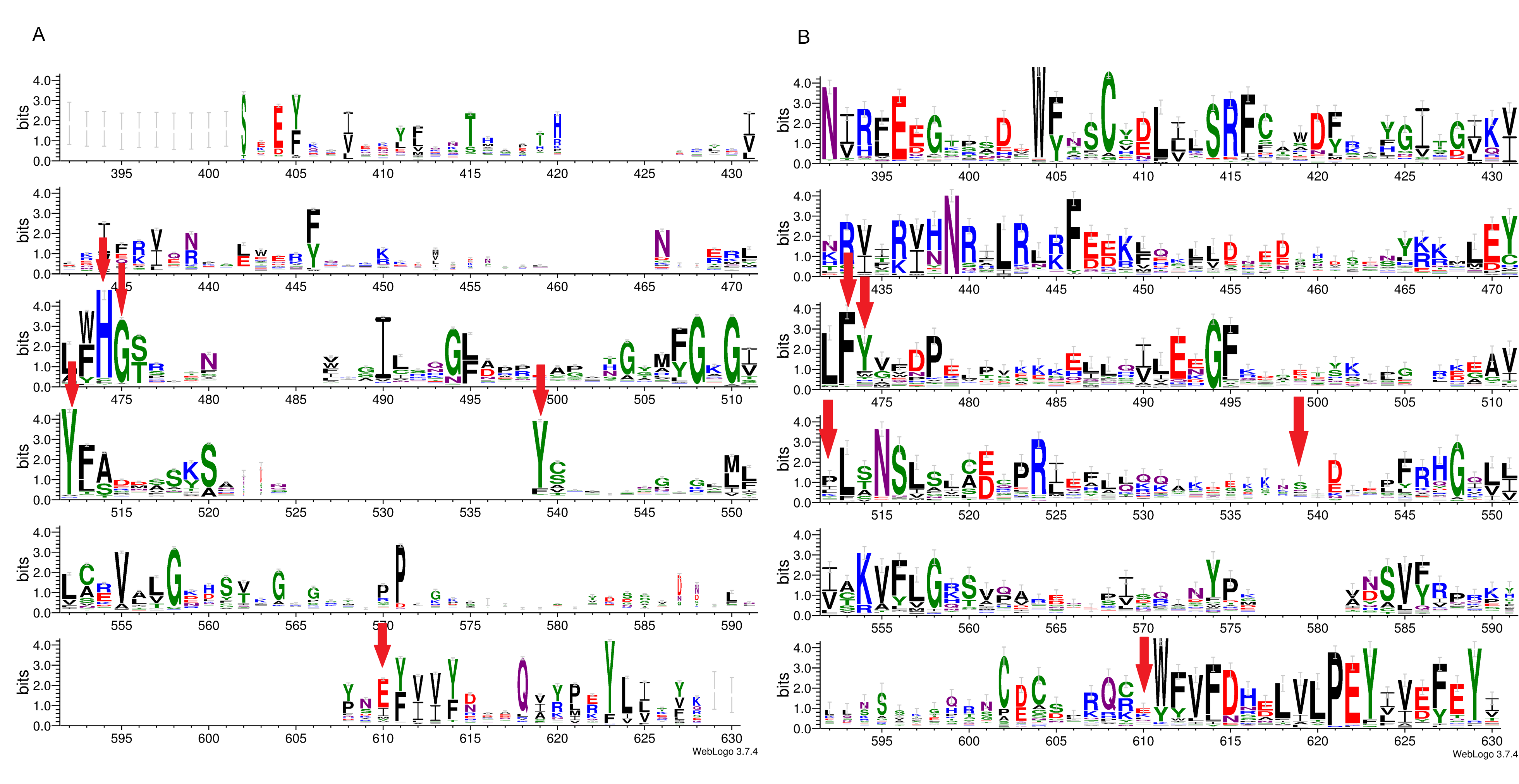

Supplement: Supplemental Information 2 — This is an alternative version of Figs. 1A and 1B, where logos are matched using a FFAS03 alignment. [file peerj-09-11051-s002.png]

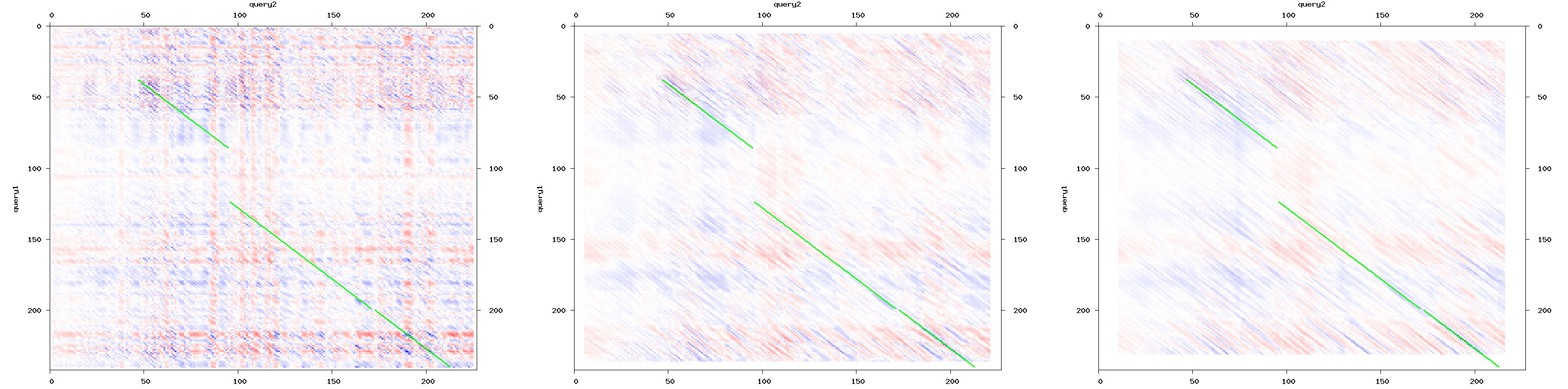

Supplement: Supplemental Information 3 — Optimal alignment returned by FFAS can also be displayed on the graph as a series of near-diagonal lines (green). An element ( M , N ) of the similarity matrix is a profile–profile similarity score of a position M in the first sequence and a position N in the second sequence. Visualization of this matrix is an M by N heat map with a color scale ranging from blue (the highest similarity between N and M ) to red (the lowest similarity). The presence of regions of high similarity not overlapping with actual alignments (green lines) suggest the presence of a sequence repeat or alternative alignment path. [file peerj-09-11051-s003.png]

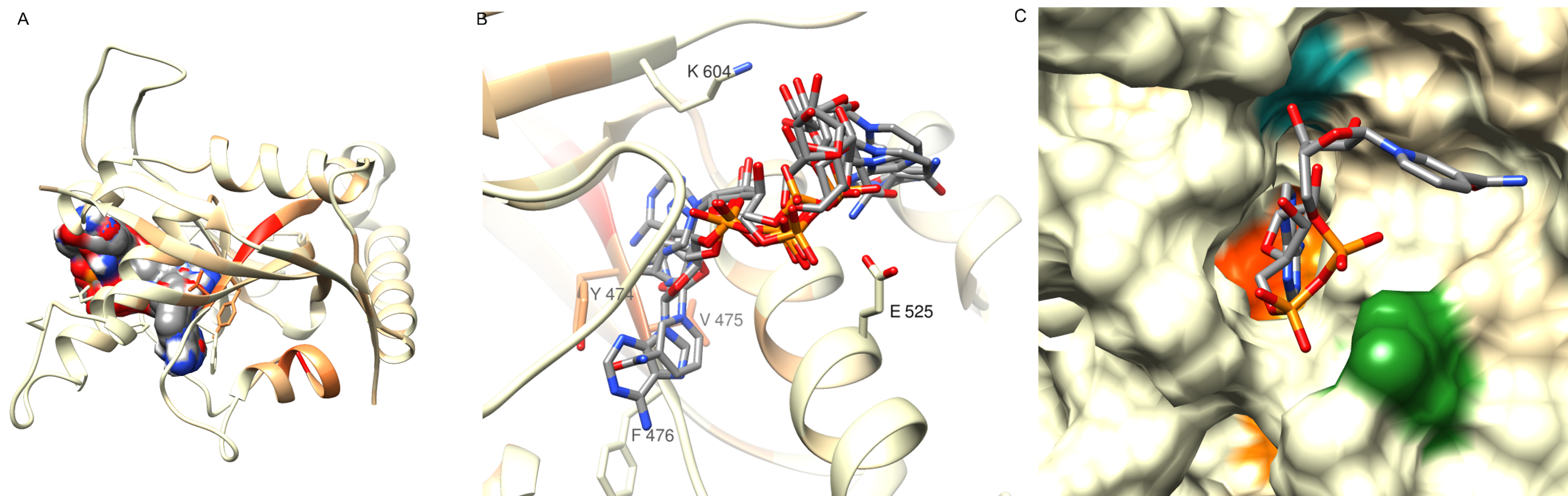

Supplement: Supplemental Information 5 — (A) Ligand poses identified by AutoDock Vina procedure (surface representation) filling the identified ligand binding pocket, see Fig. S3 G. (B) NAD poses in stick representation in the binding grove, model in ribbon representation, colored according to conservation values of sequence positions in sequence alignment of human LRRC9-ART domain homologues. PARP H-Y-Y-E tetrad residues counterparts are depicted in stick representation. (C) Best scoring NAD pose in the putative binding grove, LRRC9-ART structure in surface represenattion. Colored residues: orange - Tyr 474, Val 475, blue - Lys 604, green - Glu 525. [file peerj-09-11051-s005.png]

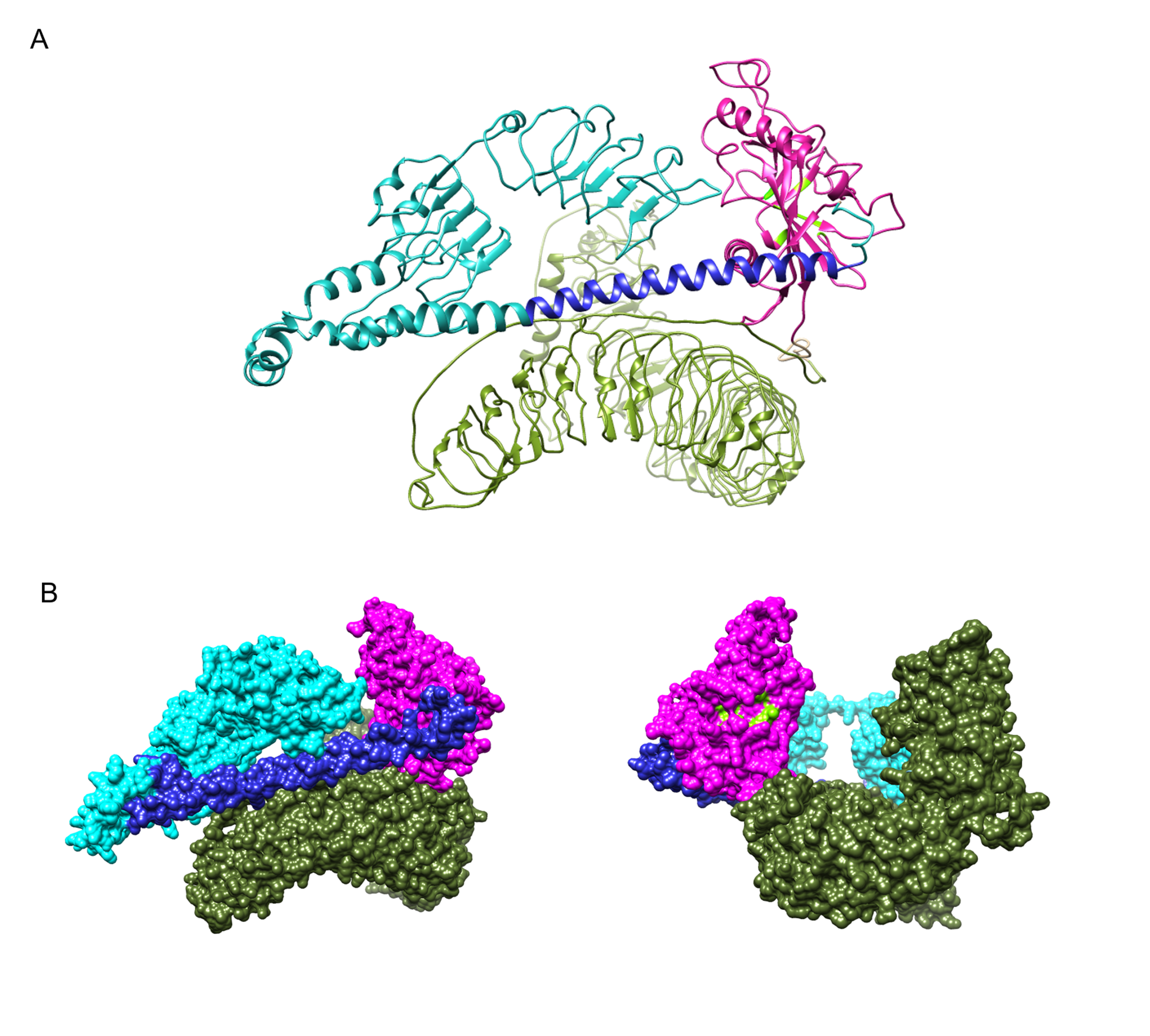

Supplement: Supplemental Information 6 — (A) ribbon representation (B) surface representation (two orientations). Magenta - LRRC9-ART domain, blue - N-terminal LRR region, olive green - C-terminal LRR region, navy blue - helical fragment between ART domain and LRR. Putative ART active site residues are marked light green. [file peerj-09-11051-s006.png]
